# How much do we really lose?—Yield losses in the proximity of natural landscape elements in agricultural landscapes

**DOI:** 10.1002/ece3.5370

**Published:** 2019-06-17

**Authors:** Larissa Raatz, Nina Bacchi, Karin Pirhofer Walzl, Michael Glemnitz, Marina E. H. Müller, Jasmin Joshi, Christoph Scherber

**Affiliations:** ^1^ Biodiversity Research Institute of Biochemistry and Biology University of Potsdam Potsdam Germany; ^2^ Leibniz‐Centre for Agricultural Landscape Research (ZALF) e.V. Müncheberg Germany; ^3^ Animal Ecology Institute of Landscape Ecology University of Münster Münster Germany; ^4^ Agroecology Department of Crop Science Georg‐August‐University Göttingen Göttingen Germany; ^5^ Plant Ecology Institute of Biology Freie Universität Berlin Berlin Germany; ^6^ Berlin‐Brandenburg Institute of Advanced Biodiversity Research (BBIB) Berlin Germany; ^7^ Institute for Landscape and Open Space Hochschule für Technik Rapperswil (HSR) Rapperswil Switzerland

**Keywords:** crop production, ecosystem services, edge effect, land sharing vs. land sparing, natural habitats, winter wheat

## Abstract

Natural landscape elements (NLEs) in agricultural landscapes contribute to biodiversity and ecosystem services, but are also regarded as an obstacle for large‐scale agricultural production. However, the effects of NLEs on crop yield have rarely been measured. Here, we investigated how different bordering structures, such as agricultural roads, field‐to‐field borders, forests, hedgerows, and kettle holes, influence agricultural yields. We hypothesized that (a) yield values at field borders differ from mid‐field yields and that (b) the extent of this change in yields depends on the bordering structure.

We measured winter wheat yields along transects with log‐scaled distances from the border into the agricultural field within two intensively managed agricultural landscapes in Germany (2014 near Göttingen, and 2015–2017 in the Uckermark).

We observed a yield loss adjacent to every investigated bordering structure of 11%–38% in comparison with mid‐field yields. However, depending on the bordering structure, this yield loss disappeared at different distances. While the proximity of kettle holes did not affect yields more than neighboring agricultural fields, woody landscape elements had strong effects on winter wheat yields. Notably, 95% of mid‐field yields could already be reached at a distance of 11.3 m from a kettle hole and at a distance of 17.8 m from hedgerows as well as forest borders.

Our findings suggest that yield losses are especially relevant directly adjacent to woody landscape elements, but not adjacent to in‐field water bodies. This highlights the potential to simultaneously counteract yield losses close to the field border and enhance biodiversity by combining different NLEs in agricultural landscapes such as creating strips of extensive grassland vegetation between woody landscape elements and agricultural fields. In conclusion, our results can be used to quantify ecocompensations to find optimal solutions for the delivery of productive and regulative ecosystem services in heterogeneous agricultural landscapes.

## 1 INTRODUCTION

During the last decades, agricultural management turned previously heterogeneous landscapes into machine‐efficient monocultures leading to a degradation and local depletion of natural landscape elements (NLEs) (Tilman et al., 2001; Vitousek et al., 1997). However, NLEs represent valuable habitats and food resources for many animals, for example, invertebrates and birds (Amy et al., 2015; Fuller & Gregory, 1995; Staley et al., 2012) delivering a range of ecosystem services such as biological pest control (Chaplin‐Kramer, O'Rourke, Blitzer, & Kremen, 2011; Woodcock et al., 2016) and pollination services (Hipólito, Boscolo, & Viana, 2018; Lindgren, Lindborg, & Cousins, 2018).

While the biodiversity value of NLEs has been frequently studied (Billeter et al., 2008), effects of NLEs on crop production are less regularly considered, although the amount of NLEs often is a “conflict zone” in the debate on biodiversity conservation versus food production in agricultural landscapes (Phalan, Onial, Balmford, & Green, 2011).

Studies investigating yield reported divergent effects depending on NLE type: Ghosh et al. (2012) showed that wheat production increased if field margins were sown with local grass species. Tschumi et al. (2015) found 10% higher yields close to flower strips, which they attributed to indirect benefits from pest control. In contrast, Sutter et al. (2018) demonstrated that ecological focus areas, such as wildflower strips, had no significant effect on oilseed rape yield, even though pollination and pest control had been increased by around 10%. From studies of hedgerows, we know that negative effects on crop yields can occur within the first meters from the field border (Kort, 1988). This effect may be caused by abiotic factors, such as shading (Esterka, 2008), but also by biotic factors, such as competition for nutrients and water (Kowalchuk & Jong, 1995), or by pests and diseases (Esterka, 2008; Thies & Tscharntke, 1999). Kort (1988), however, also showed that with increasing distance to the hedgerow, crop yields may increase above mid‐field yield values due to reduced evapotranspiration caused by wind shelter.

The relationship between distance to field border and yield was shown throughout different crop types (De Snoo, 1994; Sparkes, Jaggard, Ramsden, & Scott, 1998) and investigated in order to disentangle different factors such as weed abundance, pest incidence, and soil compaction (Boatman & Sotherton, 1988; Wilcox, Perry, Boatman, & Chaney, 2000). Still, the effect of different structures at field borders has rarely been investigated in a comparative analysis. Many authors have focused on particular structures, that is, woody or grassy landscape elements and did not incorporate other elements such as small water bodies. Moreover, most studies lacked a proper control (i.e., no field–field borders were included) to identify effects on yield arising solely from the bordering structure itself.

Here, we quantified the effects of a wide range of bordering structures, specifically agricultural roads, forest borders, hedgerows, and in‐field water bodies, on crop yields of winter wheat (*Triticum aestivum* L.) at increasing distances from the field border. As a control, we assessed yields at field‐to‐field borders. In contrast to other studies that investigated only the difference between yields at field borders and mid‐field yields (Esterka, 2008; Sklenicka & Salek, 2005), we surveyed a range of sampling points along transects starting from the border into the wheat field to detect changes in crop yield at different distances. We wanted to know (a) how far into the field yields remain notably below mid‐field yields and (b) whether the yield increase depends on the bordering structure. We hypothesized that yield losses at field borders would be stronger at tall bordering structure, such as forests, due to stronger competition effects for light, nutrient, and water.

## 2 MATERIAL AND METHODS

### 2.1 Study area

Field sampling was performed at two sites in Germany differing in climatic and edaphic conditions—the experimental farm “Klostergut Deppoldshausen” (Göttingen, Lower Saxony, Germany; 2014) and the research platform “AgroScapeLab Quillow” (Agricultural Landscape Laboratory Quillow) of the Leibniz Centre for Agricultural Landscape Research (ZALF) (Uckermark, Brandenburg, Germany; 2015–2017).

The Deppoldshausen site (5 km^2^) has a mean annual temperature of 7.7°C, annual precipitation of 645 mm (Universität Göttingen, 2009), and calcareous soils with low water‐holding capacity. On a total of 185 ha, 160 ha is used for agriculture (94% arable land and 6% permanent grassland), accompanied by 6 ha of woody habitats (forest patches and hedgerows), 5 ha of agricultural roads and ditches, and <1 ha of settlement. Half of the arable fields are managed organically; the other half is managed conventionally. On conventional fields (with 180 kg N ha^−1^ annual fertilization), winter wheat (average yields of 6.9 t/ha; measured from 2002 to 2008) is grown in rotation with winter barley and oilseed rape or depending on soil fertility with winter barley and sugar beet (Universität Göttingen, 2009).

The Quillow catchment area (250 km^2^) has a subcontinental climate with 8.7°C mean annual temperature and an annual precipitation of 475 mm (ZALF field station, Dedelow), and sandy to loamy Luvisols. The region was covered by ice during the Weichsel glaciations, so that sedimentary deposition by the glacier provided suitable conditions for an intensive agriculture with medium to high yield potentials (Stackebrandt & Manhenke, 2002). Today, local land use in the Uckermark is dominated by agriculture (62%), interspersed by forests (24%), water surfaces (5%), and settlements (5%), as well as planted hedgerows and kettle holes (small water bodies as remnants of the last ice age). 84% of the agriculturally used area accounts for arable land and 16% for permanent grasslands. The main crops on arable fields are winter cereals, oilseed rape, and silage maize cultivated on an average field size of 19 ha. Winter wheat, as the most dominant crop, yields on average 7.4 t/ha with 180–220 kg N ha^−1^ annual fertilization (measured from 2011 to 2016; Amt für Statistik Berlin‐Brandenburg, 2017, 2018) in a crop rotation most commonly after oilseed rape.

### 2.2 Study design

We established a total of 65 transects in 34 different winter wheat fields from the field border into the field over four different years at two different sites (*N* = 260). Yield samples were always taken at four distances along each transect, departing either from a NLE (forest, hedgerow, or kettle hole), an agricultural road, or a field‐to‐field border between the winter wheat field under investigation and another cereal field (Box 1, Table 1). In the following, the most distantly measured sampling point from the field border (4th distance) shall represent typical yields measured within the field and is referred to as mid‐field yield.

Box 1 **Definition of terms**

1

**Table:** 

Bordering structure	are all types of landscape elements bordering an agricultural field, such as an agricultural road, another agricultural neighboring field, a forest, hedgerow, or kettle hole
Natural landscape elements (=NLEs)	are only (semi‐) naturally occurring landscape elements such as forests, hedgerows, and kettle holes. Thus, NLE is not equal to bordering structure or transect type
Transect type	describes the different types of transects according to the bordering structure at the field border. There are five different transect types in this study, namely transects at agricultural roads, field‐to‐field borders, forests, hedgerows, and kettle holes
Transect	is our term for the linear arrangement of four distances from a field border into the field. In our design, it is a repetitive unit occurring one to three times within a winter wheat field under investigation

**Table 1:** Overview about study design for 4 years of winter wheat harvest in “Klostergut Deppoldshausen” (Lower Saxony, Germany, 2014) and in the “AgroScapeLab Quillow” (Brandenburg, Germany, 2015–2017)

	2014 Deppoldshausen	2015 Quillow	2016 Quillow	2017 Quillow
Transects starting at field borders with NLE	Forest (2) Hedgerow (2)	Forest (12)	Hedgerow (7) Kettle hole (6)	Hedgerow (8) Kettle hole (9)
Transects starting at field borders without NLE	Agricultural road (2) Field‐to‐field (2)	‐	Field‐to‐field (4)	Agricultural road (4) Field‐to‐field (7)
Distances	1 m, 4 m, 16 m, 64 m	3 m, 6 m, 30 m, 33 m	1 m, 5 m, 20 m, 50 m	1 m, 5 m, 20 m, 50 m
Sampling points	32	48	68	112

In 2014 (“Klostergut Deppoldshausen”), we established eight transects on winter wheat fields, variety *Hermann*, four of them bordering woody landscape elements: two at forests (tree height: 9 and 19 m) and two at hedgerows (tree height: 5 and 7.5 m). The remaining four transects were either situated at agricultural roads or at field‐to‐field borders (Table S1). Samples were taken at logarithmic distances (1 m, 4 m, 16 m, and 64 m); in one case, the furthest distance was shortened to 34 m because of small field size. At each sampling location (*N* = 32), we established three quadrats of 50 cm × 50 cm, where all heads of winter wheat plants (growth stage 87) were harvested by hand and threshed with a laboratory threshing machine. Seed biomass was cleaned afterward (Sample cleaner MLN, Pfeuffer), dried at 65°C for 48 hr, and weighed. For analyses, measurements were converted to grain yield in t/ha.

In 2015 (“AgroScapeLab Quillow”), six winter wheat fields were chosen adjacent to forest patches (tree height: 18–25 m). On each field, two transects were established ranging from the forest edge into the field. No transects at field‐to‐field borders were established in that year (Table S1). Samples were taken at four distances, two of which were close to the forest (3 m and 6 m) and two further away toward field center (30 m and 33 m). At all sampling points (*N* = 48), winter wheat plants (growth stage 87–89) were harvested aboveground in 1 m × 1 m plots with a sickle, threshed, and their seed biomass dried at 70°C for at least 48 hr, weighed, and converted to grain yield in t/ha.

For the field studies of 2016 and 2017 (“AgroScapeLab Quillow”), winter wheat fields were selected to be situated either adjacent to hedgerows or an in‐field kettle hole. In both years, transects at field‐to‐field borders were established, in 2017 additionally at agricultural roads (Table 1). Every transect type was situated in a different winter wheat field. Distances along transects were selected at 1 m, 5 m, 20 m, and 50 m. In 2016, seven transects started from a hedgerow (tree height: 4–13 m) and six transects from a kettle hole, each on a different winter wheat field. On four of these 13 fields, a control transect was additionally set up at a field‐to‐field border (Table S1). In 2017, 28 transects were established in eleven winter wheat fields. Each field contained a transect either departing from an agricultural road or a field‐to‐field border and at least one transect departing from a NLE, either from a hedgerow (tree height: 4–13 m) or a kettle hole. At six of eleven fields, it was possible to establish three different transect types within one field, so that we were able to harvest in total four transects at agricultural roads, seven transects at field‐to‐field borders, eight transects at hedgerows, and nine at kettle holes (Table S1). In 2016 and 2017, we harvested the total aboveground biomass of wheat plants at seed maturation (growth stage 87–89) in 1 m × 1 m plots at all sampling locations (2016: *N* = 68 and 2017: *N* = 112). After wheat plants were threshed and dried at 70°C for at least 48 hr, seed biomass was weighed and converted to yield in t/ha.

### 2.3 Statistical analyses

Responses of yield to transect types and distances were analyzed using two different kinds of analyses using R, version 3.3.1 (R Core Team, 2018): (a) with linear mixed‐effects models to qualitatively distinguish yield losses between our measured categorical distances using the package *nlme* (Pinheiro et al., 2018) and (b) with a nonlinear mixed‐effects model using the package *nlstools* (Baty et al., 2015) and assuming different competition effects per transect type at field borders as well as yields that converge asymptotically to mid‐field yields as the influence from the border vanishes.

For the full linear mixed‐effects model, we fitted the terms “transect type” (agricultural road, field‐to‐field border, forest, hedgerow, and kettle hole), categorical “distance” in spatial sequence along transects (1st, 2nd, 3rd, 4th), and their interaction term as fixed effects. Additionally, we inserted year as covariate and as random effects transects nested within fields (Tables 2 and 3). Significance levels were assessed obeying to the principle of marginality, using Wald chi‐square tests (type II), testing each term after all others, but ignoring the term's higher‐order relatives, using the *ANOVA* function (Fox & Weisberg, 2019) in the *car* package (Fox et al., 2012). In addition, we ran four smaller models (Table 4), analyzing only one transect type (forest, kettle hole, hedgerow, or agricultural road) at a time, with categorical “distance” in spatial sequence as fixed effect and transects nested within fields as random effect. Here, we used a more conservative significance level of *α* = 0.01 to adjust for the number of models analyzed. In all linear mixed‐effects models, we set the 4th distance as reference level to compare the farthest distance from the field border (referred as mid‐field yield) to those being closer to the bordering structure.

**Table 2:** Type II analysis of variance table for linear mixed‐effects model on crop yield as a function of year (2014, 2015, 2016, 2017), transect type (field‐to‐field, forest, hedgerow, kettle hole, agricultural road), categorical distance in spatial sequence along transects (1st, 2nd, 3rd, 4th), and their interaction term with the random effect term of transects nested within fields [yield ~ year + transect type * distance, random = ~1|field/transect], reference level for “distance” is the 4th distance of each year (2014 = 64 m, 2015 = 33 m, 2016/17 = 50 m) that represents mid‐field yields; reference level for “transect type” is field‐to‐field border; bold font: significant (*p* < 0.05), normal font: not significant (*p* > 0.05); *N* = 260, 65 transects in 34 fields

	*df*	*χ* ^2^	*p*‐value
Year	3	3.75	0.290
Transect type	**4**	**41.36**	**<0.001**
Distance	**3**	**135.08**	**<0.001**
Transect type:Distance	**12**	**36.82**	**<0.001**
Random term	1|field/transect		*SD* = 0.22

**Table 3:** Summary table of linear mixed‐effects model on crop yield as a function of year (2014, 2015, 2016, 2017), transect type (field‐to‐field, forest, hedgerow, kettle hole, agricultural road), categorical distance in spatial sequence along transect (1st, 2nd, 3rd, 4th), and their interaction term with the random effect term of transects nested within fields [yield ~ year + transect type * distance, random = ~1|field/transect], reference level for “distance” is the 4th distance of each year (2014 = 64 m, 2015 = 33 m, 2016/17 = 50 m) that represents mid‐field yields; reference level for “transect type” is field‐to‐field border; bold font: significant (*p* < 0.05), normal font: not significant (*p* > 0.05); *N* = 260; 65 transects in 34 fields

	Value	*SE*	*df*	*t*‐value	*p*‐value
Intercept	7.56	0.75	180	**10.09**	**<0.001**
2015 versus 2014	+1.29	0.85	29	1.51	0.142
2016 versus 2014	+0.68	0.75	29	0.90	0.374
2017 versus 2014	+0.89	0.75	29	1.20	0.242
Forest border yield change	−0.51	1.09	29	−0.47	0.642
Hedgerow yield change	−0.83	0.41	28	−2.03	0.052
Kettle hole yield change	−0.24	0.42	28	−0.58	0.567
Agricultural road yield change	+0.16	0.56	28	0.29	0.775
1st distance yield change	−1.11	0.41	180	**−2.72**	**0.007**
2nd distance yield change	−0.47	0.41	180	−1.14	0.254
3rd distance yield change	−0.56	0.41	180	−1.38	0.170
1st distance yield change at forest borders	−1.37	0.57	180	**−2.42**	**0.017**
1st distance yield change at hedgerows	−1.76	0.54	180	**−3.23**	**0.001**
1st distance yield change at kettle holes	−0.29	0.56	180	−0.53	0.600
1st distance yield change at agricultural roads	+0.14	0.73	180	0.19	0.853
2nd distance yield change at forest borders	−1.26	0.57	180	**−2.21**	**0.028**
2nd distance yield change at hedgerows	−0.74	0.54	180	−1.37	0.173
2nd distance yield change at kettle holes	−0.42	0.56	180	−0.75	0.453
2nd distance yield change at agricultural roads	−0.08	0.73	180	−0.11	0.915
3rd distance yield change at forest borders	+0.36	0.57	180	0.64	0.523
3rd distance yield change at hedgerows	+0.63	0.54	180	1.16	0.246
3rd distance yield change at kettle holes	+0.37	0.56	180	0.65	0.513
3rd distance yield change at agricultural roads	−0.12	0.73	180	−0.17	0.865

**Table 4:** Summary tables of linear mixed‐effects models on crop yield as a function of categorical distance in spatial sequence along transect (1st, 2nd, 3rd, 4th) with the random effect term of transects nested within fields per transect type [yield ~ distance, random = ~1|field/transect], reference level for “distance” is the 4th distance; bold font: significant (*p* < 0.01), normal font: not significant (*p* > 0.01)

	Value	*SE*	*df*	*t*‐value	*p*‐value
Forest (*N* = 56, 14 transects in 8 fields)					
Intercept	8.03	0.47	39	**17.25**	**<0.001**
1st distance yield change	−2.49	0.35	39	**−7.10**	**<0.001**
2nd distance yield change	−1.73	0.35	39	**−4.93**	**<0.001**
3rd distance yield change	−0.20	0.35	39	−0.57	0.572
Random effects (*SD*)	1|field = 1.11			1|field/transect = 3.29 * 10^−5^	
Hedgerow (*N* = 68, 17 transect in 17 fields)					
Intercept	7.54	0.38	48	**19.75**	**<0.001**
1st distance yield change	−2.87	0.38	48	**−7.53**	**<0.001**
2nd distance yield change	−1.21	0.38	48	**−3.18**	**0.003**
3rd distance yield change	+0.70	0.38	48	0.18	0.855
Random effects (*SD*)	1|field = 0.79			1|field/transect = 0.79	
Kettle hole (*N* = 60, 15 transects in 15 fields)					
Intercept	8.05	0.40	42	**20.07**	**<0.001**
1st distance yield change	−1.41	0.42	42	**−3.38**	**0.002**
2nd distance yield change	−0.89	0.42	42	−2.14	0.038
3rd distance yield change	−0.20	0.42	42	−0.47	0.638
Random effects (*SD*)	1|field = 0.75			1|field/transect = 0.75	
Agricultural road (*N* = 24, 6 transects in 6 fields)					
Intercept	7.96	0.39	15	**20.26**	**<0.001**
1st distance yield change	−0.98	0.36	15	−2.74	0.015
2nd distance yield change	−0.55	0.36	15	−1.53	0.146
3rd distance yield change	−0.69	0.36	15	−1.93	0.072
Random effects (*SD*)	1|field = 0.52			1|field/transect = 0.52	

For the nonlinear mixed‐effects model, we implemented a self‐starting function with varying yield values at field border (*Y_c_*), a fixed rate (*c*) at which yields putatively increase as well as varying mid‐field yields (*Y_m_*) at which the curves converge asymptotically per transect type:

(1) $$Y\left(x\right)∼e^{-cx}*\left(Y_{c}-Y_{m}\right)+Y_{m}$$

*Y_c_* and *Y_m_* were modeled as functions of transect type with random effect of transects nested within fields to allow the function to vary at field border and at mid‐field yields per transect type (Table 5). Unfortunately, the low number of sampling points did not allow us to vary all three parameters per transect type and we thus fixed the exponential decay rate at *c* = 0.1 m^−1^ after visual inspection.

**Table 5:** Summary table of nonlinear mixed‐effects model on crop yield as self‐starting function with varying yield values at field border (*Y_c_*), a fixed rate (*c*) at which yields increase as well as varying mid‐field yields (*Y_m_*) at which yields converge asymptotically per transect type (Equation 1). *Y_c_* and *Y_m_* were modeled as functions of transect type with random effect of transects nested within fields; reference level is field‐to‐field; bold font: significant (*p* < 0.05), normal font: not significant (*p* > 0.05); *N* = 260; 65 transects in 34 fields

	Value	*SE*	*df*	*t*‐value	*p*‐value
*c*	0.12	0.02	185	**5.63**	**<0.001**
*Y_c_* (intercept)	7.15	0.46	185	**15.46**	**<0.001**
*Y_c_* (forest)	−2.49	0.74	185	**−3.37**	**<0.001**
*Y_c_* (hedgerow)	−2.73	0.57	185	**−4.81**	**<0.001**
*Y_c_* (kettle hole)	−0.71	0.57	185	−1.23	0.221
*Y_c_* (agricultural road)	+0.28	0.78	185	+0.37	0.712
*Y_m_* (Intercept)	8.08	0.27	185	**29.69**	**<0.001**
*Y_m_* (forest)	−0.01	0.46	185	−0.02	0.988
*Y_m_* (hedgerow)	−0.38	0.28	185	−1.36	0.177
*Y_m_* (kettle hole)	−0.09	0.27	185	−0.32	0.749
*Y_m_* (agricultural road)	+0.24	0.38	185	+0.63	0.527
Random effect (*b*)	1|field = 1.34 * 10^−7^				
Random effect (*Y_c_*)	1|field = 0.93			1|field/transect = 1.17	
Random effect (*Y_m_*)	1|field = 0.89			1|field/transect = 0.19	

With this function at hand, we were able to quantify the distance at which 95% of mid‐field yield per transect type were reached (Figure 3 and Table 6):

(2) $$\begin{array}{c} 0.95\;Y_{m}=e^{-cx_{95\%}}*\left(Y_{c}-Y_{m}\right)+Y_{m}\\ x_{95\%}=-\ln\left(\frac{0.05}{1-\frac{Y_{c}}{Y_{m}}}\right)c^{-1} \end{array}$$

In addition, we calculated absolute yield loss (*L*) in kg per meter field border by subtracting each surface integral (*A*) from the total area under the asymptote until 95% of mid‐field yield:

(3) $$\begin{array}{c} F\left(x\right)=\int_{0}^{x_{95\%}}e^{-cx}*\left(Y_{c}-Y_{m}\right)+Y_{m}\\ A=-\frac{1}{c}e^{-cx_{95\%}}*\left(Y_{c}-Y_{m}\right)+Y_{m}x_{95\%}+\frac{1}{c}*\left(Y_{c}-Y_{m}\right)\\ L\;\left[\text{kg/m}\right]=\left(Y_{m}x_{95\%}-A\right)/10 \end{array}$$

**Table 6:** Calculated values for the distance [m] (*x*_95%_) at which 95% of mid‐field yields were reached (Equation 2) and absolute [kg/m] as well as relative [%] yield losses that occurred from the field border until *x*_95%_ compared to no field border (Equation 3). Yield loss is calculated as subtraction of the surface integral (*A*) bounded between 0 and *x*_95%_ of the total area of mid‐field yields (*Y_m_*) multiplied by the distance (*x*_95%_) at which 95% of mid‐field yields are reached; all values are given per transect type

	*x*_95%_ [m]	Losses [kg/m]	Losses [%]
Agricultural road	6.32	0.39	7.48
Field‐to‐field	6.93	0.44	7.80
Forest	17.79	2.51	17.47
Hedgerow	17.85	2.41	17.54
Kettle hole	11.28	0.96	10.60

With this, we could quantify relative yield loss over the area from the field border until the distance where yield reaches 95% of mid‐field yield per meter field border.

## 3 RESULTS

**Figure 1:**
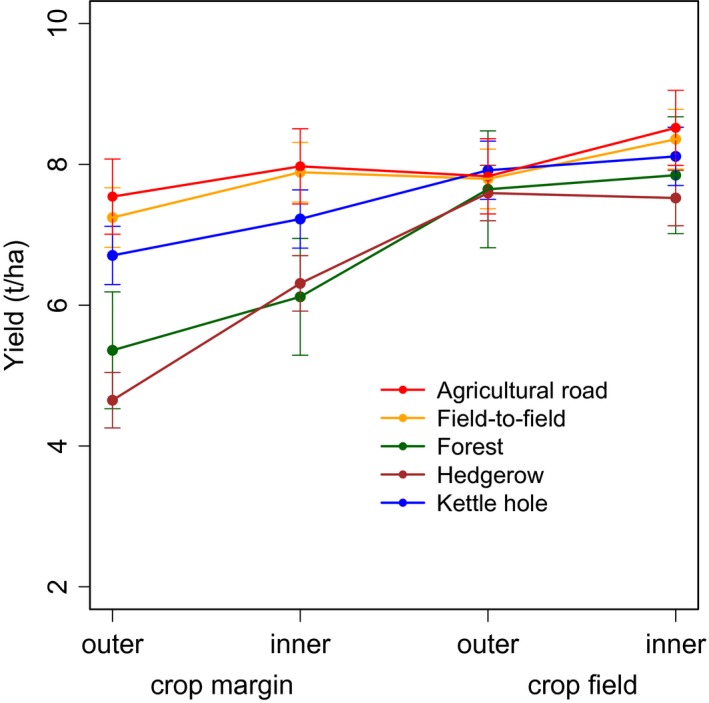
Winter wheat yield, measured as seed biomass [t/ha], along transects departing from the field border toward the field center measured in four categorical distances in spatial sequence (1st = outer crop margin, 2nd = inner crop margin, 3rd = outer crop field, 4th = inner crop field) over four years of investigation (2014 = 1 m, 4 m, 16 m, 64 m; 2015 = 3 m, 6 m, 30 m, 33 m; 2016 and 2017 = 1 m, 5 m, 20 m, 50 m) adjacent to agricultural roads (*N* = 24; red), to field‐to‐field borders (*N* = 52; orange), to forest borders (*N* = 56; green), to hedgerows (*N* = 60; brown), and to kettle holes (*N* = 60; blue); for detailed attributions of transect types per year, see Table S1. Values are depicted as fitted values with confidence intervals of 95% taken from the linear mixed‐effects model with crop yield as a function of year, transect type, categorical distance, and their interaction term with the random effect term of transects nested within fields. *N* = 260, 65 transects in 34 fields.

We measured an average winter wheat yield of 6.42 ± 1.14 t/ha (mean ± standard deviation) seed biomass in “Klostergut Deppoldshausen” (2014), as well as 7.23 ± 1.67 t/ha (2015), 6.99 ± 1.61 t/ha (2016), and 7.49 ± 1.77 t/ha (2017) in the “AgroScapeLab Quillow.” Generally, winter wheat yields increased from the field border into the agricultural field (*χ* ^2^(3) = 135.1, *p* < 0.001; Figure 1; Table 2). Adjacent to the investigated bordering structures, we observed a yield reduction compared to yields measured farthest from it at the 1st to 4th distance of 1.11 ± 0.41 t/ha (13%) at field‐to‐field borders (*t*_180_ = −2.7, *p* < 0.01; Table 3); 2.49 ± 0.35 t/ha (32%) at forest borders (*t*_39_ = −7.1, *p* < 0.001; Table 4); 2.87 ± 0.38 t/ha (38%) at hedgerows (*t*_48_ = −7.5, *p* < 0.001); 1.41 ± 0.42 t/ha (17%) at kettle holes (*t*_42_ = −3.4, *p* < 0.01); and 0.98 ± 0.36 t/ha (11%) at agricultural roads (*t*_15_ = −2.7, *p* < 0.05)). This significant yield loss persisted adjacent to forest borders (*t*_39_ = −4.9, *p* < 0.001) and hedgerows (*t*_48_ = −3.2, *p* < 0.01) when comparing the 2nd and 4th distances from field border. At the 3rd sampling point, all yield differences were negligible between investigated transect types (Figure 1). Yield losses varied between transect types (*χ* ^2^(12) = 36.8, *p* < 0.001; Figure 1; Table 2), especially when comparing field‐to‐field borders with woody landscape structures: Forest borders affected yields negatively compared to a neighboring agricultural field at the 1st and 2nd distance (*t*_180_ [1st] = −2.4, *p* < 0.05; *t*_180_ [2nd] = −2.2, *p* < 0.05; Table 3). Yields at hedgerows differed only within the 1st distance to yields measured at field‐to‐field borders (*t*_180_ = −3.2, *p* < 0.01). However, we found that yield losses adjacent to kettle holes were similar to those observed next to another agricultural field (*t*_180_ = −0.5, *p* > 0.5).

**Figure 2:**
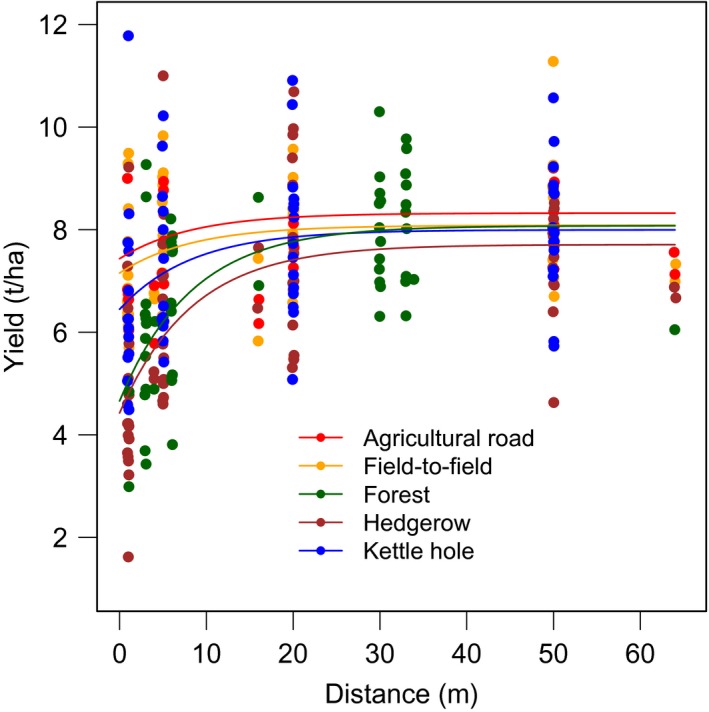
Winter wheat yield, measured as seed biomass [t/ha], along transects departing from the field border toward the field center fitted in a nonlinear mixed‐effects model as a self‐starting function with varying yield values at field border (*Y_c_*), a fixed rate (*c*) at which yields increase as well as varying mid‐field yields (*Y_m_*) at which yields converge asymptotically per transect type (Equation 1). *Y_c_* and *Y_m_* were modeled as functions of transect type with random effect of transects nested within fields. Transect types were agricultural roads (*N* = 24; red), field‐to‐field borders (*N* = 52; orange), forest borders (*N* = 56; green), hedgerows (*N* = 60; brown), and kettle holes (*N* = 60; blue); for detailed attributions of transect types per year, see Table S1. *N* = 260, 65 transects in 34 fields

**Figure 3:**
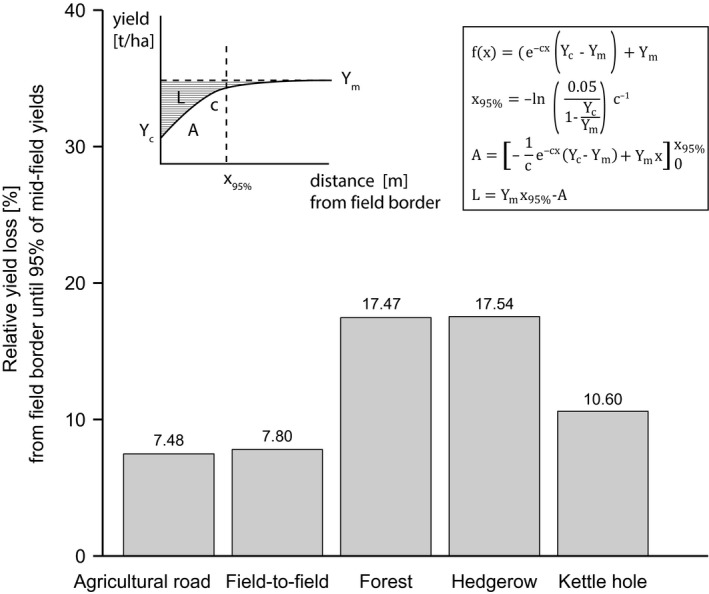
Relative yield loss [%] from the field border until 95% of mid‐field yields are reached per transect type (agricultural road, field‐to‐field border, forest, hedgerow, and kettle hole) with a simplified sketch of the relationship between distance [m] from field border and yield [t/ha] as a nonlinear function with varying yield values at field border (*Y_c_*), a fixed rate (*c*) at which yields increase as well as varying mid‐field yields (*Y_m_*) at which the yields converge asymptotically per transect type (Equation 1) where yield loss (*L*) is calculated as subtraction of the surface integral (*A*) bounded between 0 and *x*_95%_ of the total area of mid‐field yields (*Y_m_*) multiplied by the distance (*x*_95%_) at which 95% of mid‐field yields are reached (Equations 2 and 3)

Fitting the yield increase from the field border into the field with a nonlinear asymptotic function (Equation 1) revealed a similar pattern (Figure 2). At forest borders and hedgerows, winter wheat yields proximate to the bordering structure (*Y_c_*) were significantly lower than at field‐to‐field borders (forest: *t*_185_ = −3.4; *p* < 0.001; hedgerow: *t*_185_ = −4.8; *p* < 0.001; Table 5). Adjacent to kettle holes, however, no yield reduction could be observed compared to a neighboring agricultural field (*t*_185_ = −1.2; *p* > 0.1). The effect of a bordering structure vanished further into the field and transect type had no influence on mid‐field yields (*Y_m_*). To specify how far this yield reduction lasted into the field, and how this depended on transect type, we calculated the distance until 95% of mid‐field yields are reached using the asymptotic function (Figure 3). We found that agricultural roads reduced yields up to 6.3 m into the field (Table 6). Up to this distance, the farmer loses 7.5% per meter agricultural road of the yield that could have been achieved without any field border. Field‐to‐field borders affected yields up to 6.9 m for 95% mid‐field yields with a loss of 7.8% per meter field border. Woody landscape elements showed the most far‐ranging effect of the investigated transect types with yield losses of 17.5% until 95% of mid‐field yields. These yields were reached for both transect types after 17.8 m from forest borders or hedgerows. Per meter kettle hole, 10.6% of seed biomass was lost until 11.3 m, where the benchmark of 95% of mid‐field yields is met (Figure 3, Equation 2 + 3 and Table 6).

## 4 DISCUSSION

We investigated the effect of different natural landscape structures (NLE: forests, hedgerows, and kettle holes) on winter wheat yields comparing yields along transects from NLEs as well as from agricultural roads and field‐to‐field borders into agricultural fields across two German regions. At all transect types, yields next to the field border (1–3 m) were reduced compared to mid‐field yields. However, depending on the bordering structure, yield differences vanished at varying distances: For woody landscape elements, yield loss was still considerably high at the 2nd measured distance (4–6 m) and reached 95% of mid‐field yields only after 17 m. In contrast, yield reduction adjacent to kettle holes did not differ compared to field‐to‐field borders. Here, yields converged already within 11 m to 95% of seed biomass values measured within the field.

### 4.1 Negative effects of natural landscape elements close to the border

A potential explanation for lower yields close to NLEs could simply be that farmers do not apply chemical inputs such as fertilizers, plant promoters, and plant protection in full amounts close to field borders. Law restricts the application of fertilizers and pesticides at field borders in Germany. For plant protection issues, distance restrictions to any NLE neighboring agricultural fields are set by EU regulation (No. 1107/2009) and detailed by the German plant protection law (Deutscher Bundestag, 2012). The restrictions vary regarding the active agents of the plant protection measure, the kind of neighboring NLE, and the amount of NLEs in the surrounding landscape (Bayerische Landesanstalt für Landwirtschaft, 2018). Still, most restrictions range between 5 and 20 m, agreeing with the distances of noticeable yield loss next to our investigated NLEs. Adjacent to water bodies, farmers are additionally restricted by regulations for fertilization to maintain a minimum spraying distance depending on fertilizer types, spreading technique, and slope of the field border (BMEL, 2017). Therefore, the reduced nutrient and plant protection input may contribute to lower yields close to these landscape elements.

Another factor that potentially causes yield losses at field borders is soil compaction due to turning of machinery (Boatman & Sotherton, 1988; Wilcox et al., 2000). This occurs mostly where tram tracks are perpendicular to field border and therewith to the bordering structure. As we did not control for this factor originally, some of our transects were aligned parallel to tram tracks and thus situated at field borders where machinery turns. However, a smaller share of our transects was situated at field borders with turning edges and these did not result into lower yields compared to nonturning edges (Figure S1, Tables S2 and S3). Thus, we cannot conclusively show that turning edges had an influence on our results.

Previously, NLEs had been hypothesized to increase crop yield, for example, because of overall enhanced biodiversity and biocontrol (Tschumi et al., 2015). While we did not assess predator abundance or biocontrol efficiency in this study, it is likely that predator–prey interactions (e.g., with pest antagonists) were not an important mechanism to explain crop yields in the intensively managed winter wheat fields in our study that were treated with pesticides (Tscharntke et al., 2016), albeit less intensively at field margins. Insect pest populations may even have benefitted from the presence of a NLE by using it as habitat or food resource when the annual crops were harvested (Blitzer et al., 2012). Thus, reduced crop yields close to NLEs could be caused by a combination of reduced chemical inputs and increased pest pressure—but the exact mechanisms remain to be tested in future studies.

However, the most cited explanation for yield reduction close to NLEs is shading, especially adjacent to tall vegetation structures (Burgess, Incoll, Corry, Beaton, & Hart, 2004; Esterka, 2008; Kort, 1988). Lyles, Tatarko, and Dickerson (1984) showed yield reductions within winter wheat fields up to a distance equivalent to twice the height of the trees, with an average decrease by 31% compared to mid‐field yield. Shade may also preserve soil moisture next to the NLE, resulting in a higher risk of crop‐pathogen infection (Müller et al., 2016). Müller et al. (2016) reported that abundances of fungal wheat pathogens were correlated with higher soil moisture.

### 4.2 Different natural landscape elements—different effects

There are hardly any data available showing the impact of different NLE types on yield. In consistence with our second hypothesis, we have shown that the type of bordering structure influenced the relationship between yield and distance to the field border. For kettle holes, yield reduction was not as severe as for woodlands; already after 11.3 m, 95% of mid‐field yields were obtained. Hence, only 5 m later than the observed distance at which 95% of mid‐field yields are reached close to fieldto‐field borders. Still, yields measured closest to kettle holes were affected negatively in both years of investigation (2016 and 2017), probably because of reduced chemical plant protection and fertilization close to water bodies. Nonetheless, seed biomass values adjacent to kettle holes were statistically indistinct from yields measured at field‐to‐field border. Especially in 2016, we observed a trend to higher yield at 20 m distance to the kettle hole compared to field‐to‐field borders, suggesting that in years with low precipitation (2016:422 mm; ZALF field station, Dedelow) these natural water islands can act as water supplier for the crop plant (Figure S2). Thus, yield losses near kettle holes may be negligible and the value of this NLE for biodiversity conservation and regulative services for the crop may not incur any economic losses for the farmer.

Yield reductions observed close to forest borders and hedgerows might be driven in large part by shading from trees (Burgess et al., 2004) or by tree roots entering arable land belowground conferring competition for water and nutrients (Huber, Schmid, Birke, & Hülsbergen, 2013). Regarding shading effects, as we did not explicitly choose our transects according to their exposure, we unfortunately could not clearly show in our analyses that yields at south‐oriented transects were at an advantage compared with those measured at north‐orientated transects (Figure S3, Table S4). We observed only a trend at hedgerows that was not confirmed by forest borders. Among others, Sklenicka and Salek (2005) reported crop yield losses to become insignificant at a distance between twice to three times the height of the adjacent trees. In 2015, the investigated forest borders ranged between 18‐m and 24‐m tree height and most of our investigated hedgerows in the “AgroScapeLab Quillow” in 2016 and 2017 were 4 m to 13 m tall. Still, significant yield losses could only be detected for the 1st and 2nd distance (4–6 m) at forest and hedgerows compared to the respective mid‐field yields. At the 3rd distance (16–30 m), yields were indistinct to yields measured at field‐to‐field borders as well as to mid‐field yields. The nonlinear function we applied to capture the increase in yields away from the field border revealed that already at a distance of 17.8 m from both woody landscape elements 95% of mid‐field yields could be reached. These results indicate that in our study yield losses are not as severe as reported in former literature (Esterka, 2008; Kort, 1988; Lyles et al., 1984; Sklenicka & Salek, 2005).

In addition, we could observe trends to higher yields at 20 m distance from woody landscape elements compared to mid‐field yields in 2014 and 2016 (Figure S2). It is therefore likely that woodlands have also provided a positive (potentially sheltering) effect for the crop that had been outside the trees' shading scope. There, not being light‐limited, the crop could have benefitted from reduced evapotranspiration as woodlands are known to act as wind barriers and can lower wind speed to distances from twice to four times the height of the trees (Kowalchuk & Jong, 1995; Peter & Bozsik, 2009). These shelter effects can be particularly relevant in drought years or in future drier scenarios under climate change, where maintained soil moisture becomes highly valuable (Thaler, Eitzinger, Trnka, & Dubrovsky, 2012). Accordingly, in wet years, the shelter effect was shown to be less pronounced or even absent (Bruckhaus & Buchner, 1995; Kowalchuk & Jong, 1995). This pattern can also be observed in our data (Figure S2), as this yield peak was more evident in the dry year of 2016 (422 mm of precipitation) compared to 2017 (755 mm of precipitation, ZALF field station, Dedelow). We tried to capture the shelter effect in our data by fitting a biexponential function to our data:

(4) $$\text{yield}∼\left(e^{-cx}*\left(Y_{c}-Y_{m}\right)+Y_{m}\right)*\left(e^{-sx}*\left(Y_{s}-Y_{m}\right)+Y_{m}\right)$$

Unfortunately, we were unable to achieve convergence due to scarcity of data points given the larger number of free parameters. The intended function incorporates detrimental competition effects that decrease into the field at rate c and beneficial effects of shelter that decrease into the field as well at a somewhat lower rate s. In addition, yield values at field borders affected only by the shelter effect (*Y_s_*), thus excluding competition, should be higher than mid‐field yields (*Y_m_*) in contrast to those affected from only the competition effect (*Y_c_*) being lower than mid‐field yields. We would like to encourage further investigations on yield losses at different bordering structures to design their studies based on these two effects.

### 4.3 Management recommendations

In order to profit from the beneficial effect of woody landscape elements (even though yields are lowered proximate to it), we propose cutting hedgerows on a regular basis. Such management regimes are already in place in Switzerland, where hedgerow height is limited by a compulsory rotational trimming management at least every 8 years (Federal Office of Agriculture FOAG Switzerland, 2018). Such standards could reduce disadvantages of shading and competition for nutrients and water for the proximate crop plants, while the advantages of, for example, reduced evapotranspiration and pest control would be kept.

Another measure could be to design more efficient field borders at woody landscape elements by combining different NLEs to strengthen advantages for biodiversity and the provision of regulating and supporting ecosystem services such as water regulation and pest control. In particular, an option could be to keep a broad fringe of extensive grassland vegetation at borders between a forest or hedgerow and an agricultural field as recommended by Berger et al. (2011) and implemented by Swiss agricultural directives. Pywell et al. (2015) already showed that the creation of grassy wildlife‐friendly habitats at field borders not only increases pollinator abundance but also leads to increased yields proximate to it. Moreover, they demonstrated that removing 8% of the farmland at field borders for those habitats can balance overall yield losses and pay off already after five years of maintenance. The width of such habitats should cover the zone of severe yield losses depending on the bordering structure, but ensure that crop production benefits from the positive effect of, for example, woody landscape elements after release from competition. As herbaceous landscape elements do not result in significant yield losses (Sutter et al., 2018; Tschumi et al., 2015), a herbaceous strip with a diverse mix of short‐lived and perennial native plants as applied in Pywell et al. (2015) could be a perfect habitat to obtain multiple advantages: No severe yield loss at field borders, combined with further positive effects from different habitats—wind shelter, and erosion control gained by the woody landscape element and high pollinators and pest–predator abundances obtained by the herbaceous landscape element.

## 5 CONCLUSIONS

Natural landscape elements in agro‐ecosystems are crucial, not only for biodiversity conservation but also for promoting regulating and supporting services such as water regulation and pest control. Our findings can add to the debate on economic gains and losses from specific NLEs as we quantified yield losses depending on the bordering structure. In particular, we showed that the effect of NLEs on crop yields varies between NLE types. They can be negligible (as for kettle holes), negative close to woody landscape elements, and even slightly positive at more than twice the distance of tree height from hedgerows or forest borders. We therefore recommend aligning a second NLE with lower vegetation (e.g., an herbaceous strip) in‐between the field border and a woody landscape element to maintain their longer‐ranged positive effects and buffer their short‐ranged negative effects by naturally provided ecosystem services that can benefit farmers at low economic costs. Future studies should focus on finding the optimal balance between the provisioning service for crop production and sustainable land management, where NLEs are an important part of agricultural landscapes.

## CONFLICT OF INTEREST

None declared.

## AUTHOR CONTRIBUTIONS

LR, KPW, JJ and CS conceived the ideas and designed the methodology; LR, NB, KPW, MM, and MG collected the data; LR and CS analyzed the data; and LR led the writing of the manuscript. All authors contributed critically to the drafts and gave final approval for publication.

## Data Availability

Data are registered at ZALF Open Research Data (https://doi.org/10.4228/ZALF.DK.100).
