# Supplementary figures and images for: How much do we really lose?—Yield losses in the proximity of natural landscape elements in agricultural landscapes
Source: Ecol Evol. 2019 Jun 17;9(13):7838–48. doi: 10.1002/ece3.5370 (PMC6635954; doi:10.1002/ece3.5370)

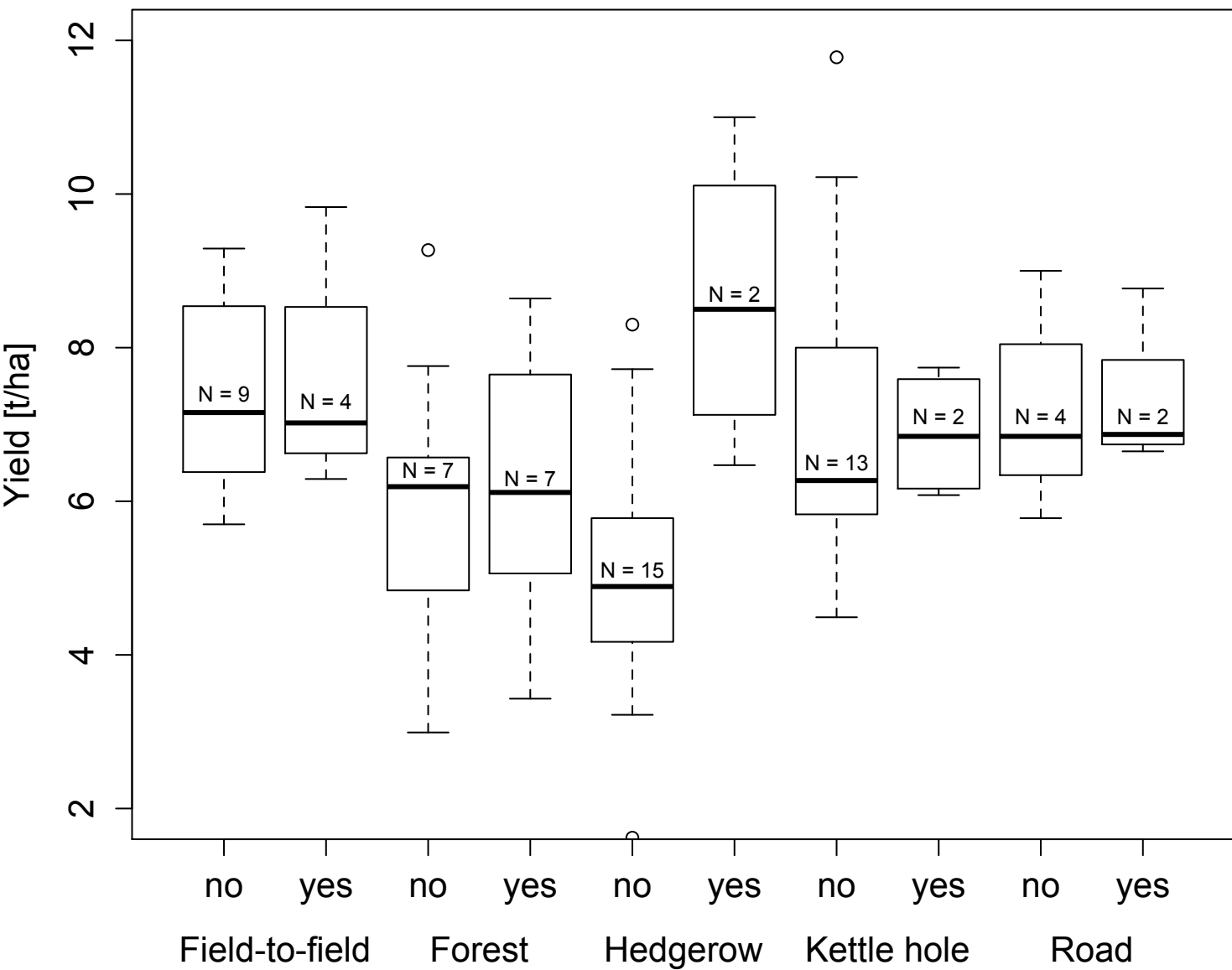

Supplement: Supplementary file 1 [file ECE3-9-7838-s001.pdf]

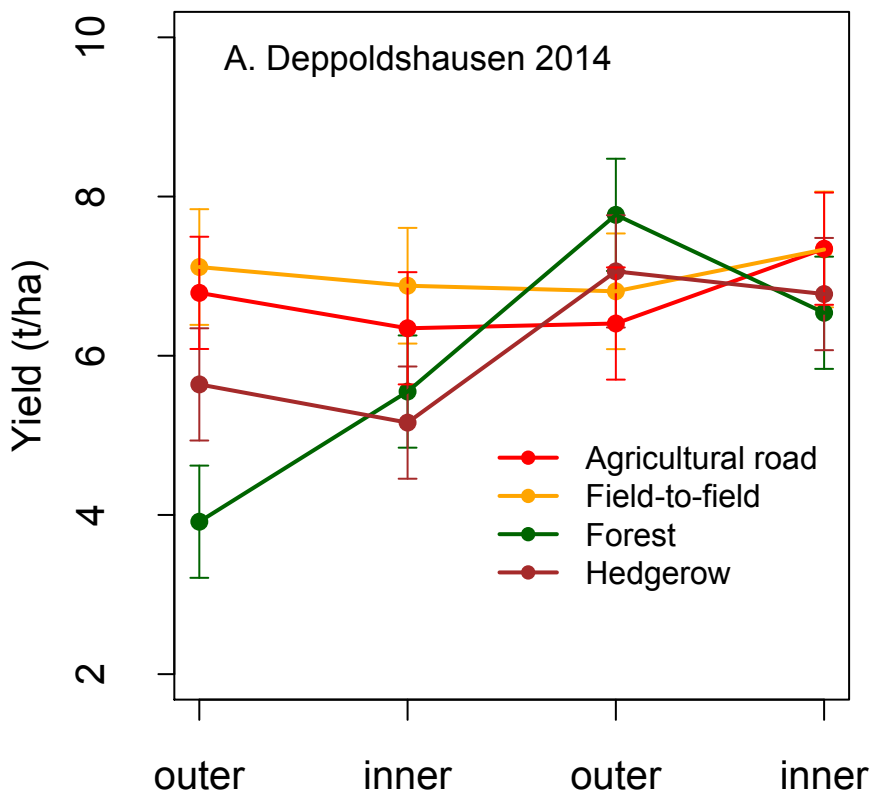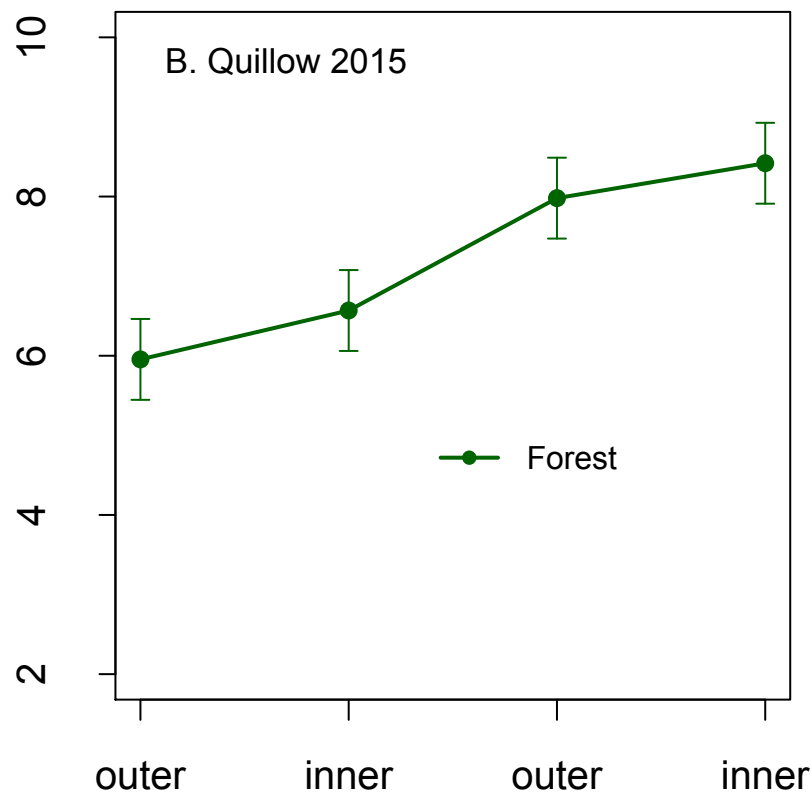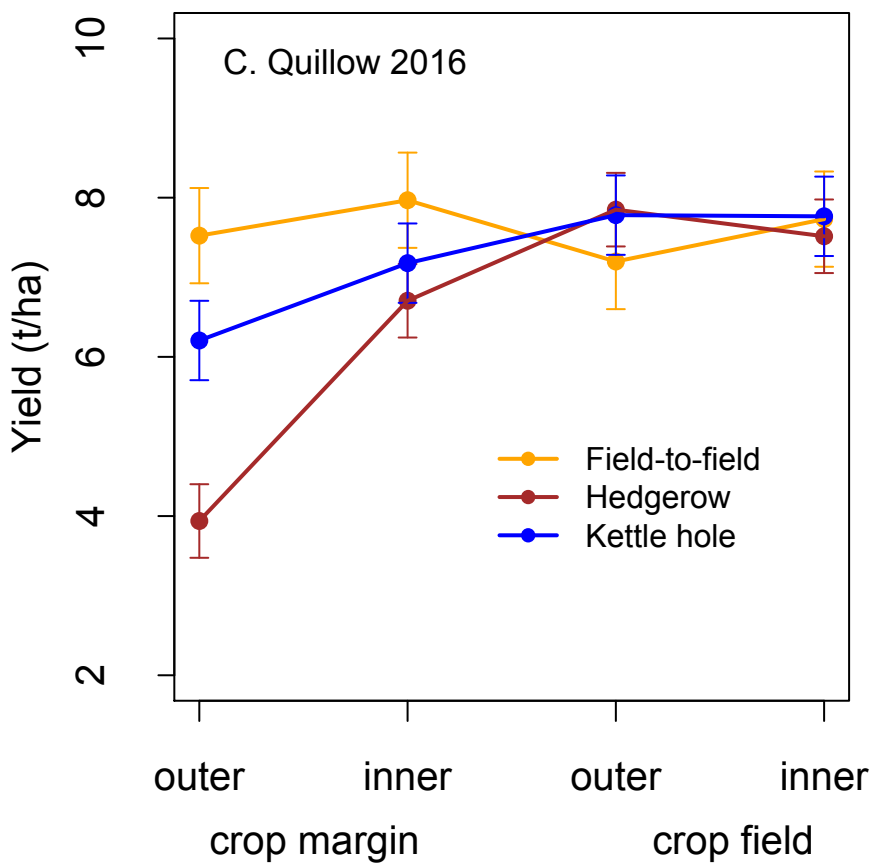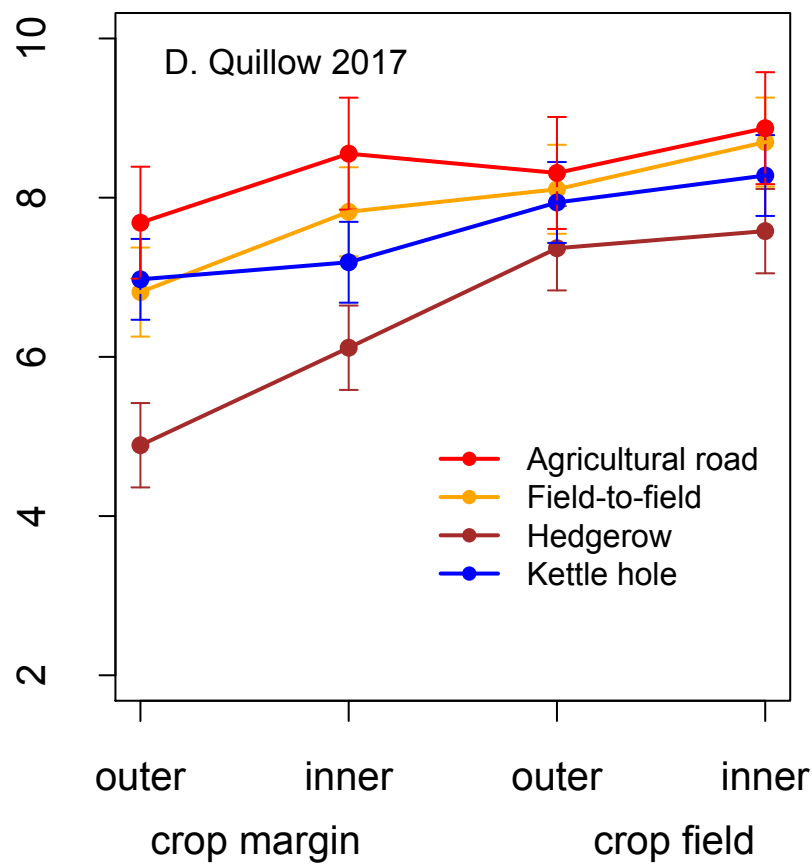

Supplement: Supplementary file 2 [file ECE3-9-7838-s002.pdf]

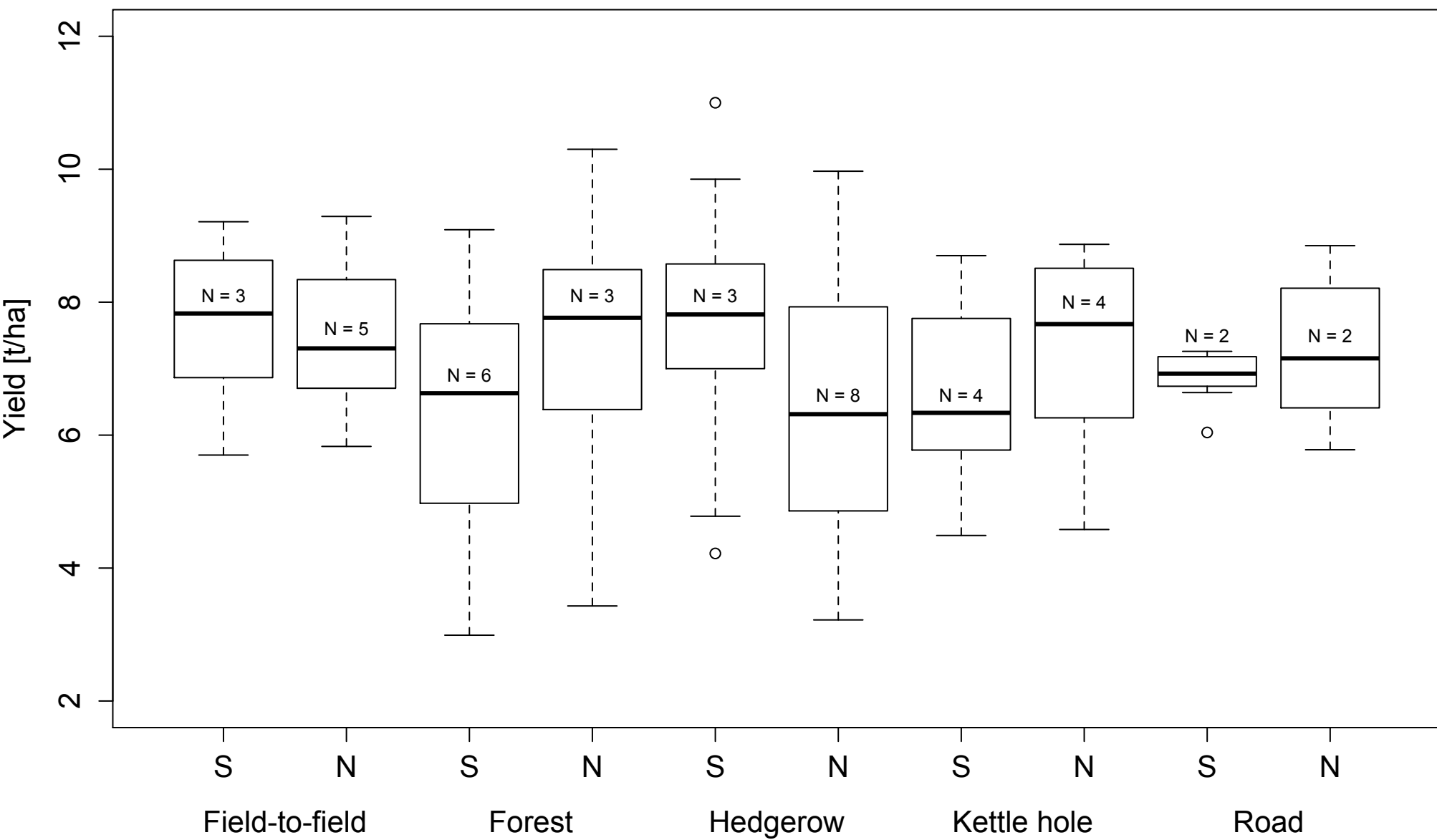

Supplement: Supplementary file 3 [file ECE3-9-7838-s003.pdf]
