# Supplementary material for: How much do we really lose?—Yield losses in the proximity of natural landscape elements in agricultural landscapes
Source: Ecol Evol. 2019 Jun 17;9(13):7838–48. doi: 10.1002/ece3.5370 (PMC6635954; doi:10.1002/ece3.5370)
Supplement: Supplementary file 4 [file ECE3-9-7838-s004.docx]

**Appendix**

**Figure Legends**

Figure S1: Boxplots of winter wheat yield [t ha^-1^] at field borders (1^st^ and 2^nd^ distance) per transect type (field-to-field, forest, hedgerow, kettle hole and agricultural road) divided in either transects without turning edge (no) or with turning edge (yes) according to visual inspections of satellite images; N is given as number of transects, each with two sampling points, one at the 1^st^ and the other at the 2^nd^ distance.

Figure S2: Winter wheat yield, measured as seed biomass [t ha^-1^], along transects departing from the field border towards the field centre measured in four categorical distances in spatial sequence (1^st^ = outer crop margin, 2^nd^ = inner crop margin, 3^rd^ = outer crop field, 4^th^ = inner crop field) per year:

1. ‘Klostergut Deppoldshausen’ (2014) adjacent to agricultural roads (N = 8; red), to field-to-field borders (N = 8; orange), to forest borders (N = 8; green) and to hedgerows (N = 8; brown) whereby categorical distances in spatial sequence are 1 m, 4 m, 16 m, and 64 m.
2. ‘AgroScapeLab Quillow’ (2015) adjacent to forest borders (N = 48; green) whereby categorical distances in spatial sequence are 3 m, 6 m, 30 m, and 33 m.
3. ‘AgroScapeLab Quillow’ (2016) adjacent to field-to-field borders (N = 16; orange), to hedgerows (N = 28; brown) and to kettle holes (N = 24; blue) whereby categorical distances in spatial sequence are 1 m, 5 m, 20 m, and 50 m.
4. ‘AgroScapeLab Quillow’ (2017) adjacent to agricultural roads (N = 16; red), field-to-field borders (N = 28; orange), to hedgerows (N = 32; brown) and to kettle holes (N = 36; blue) whereby categorical distances in spatial sequence are 1 m, 5 m, 20 m, and 50 m.

Values are depicted as fitted values with confidence intervals of 95% taken from linear mixed-effects models with crop yield as a function of transect type, categorical distance and their interaction term with the random effect term of transects nested within fields.

Figure S3: Boxplots of winter wheat yield [t ha^-1^] per transect type (field-to-field, forest, hedgerow, kettle hole and agricultural road) divided in either transects north (N, NW, N, NE) or south (S, SE, S, SW) orientated from the field border into the field. N is given as number of transects, each with four sampling points.

**Tables**

Table S1: Detailed characteristics of fields and transects in four years winter wheat harvest in ‘Deppoldshausen’ (Lower Saxony, Germany, 2014) and in the ‘AgroScapeLab Quillow’ (Brandenburg, Germany, 2015-2017) regarding transect type (agricultural road, field-to-field border, forest, hedgerow and kettle hole), turning edge (‘Yes’ or ‘No’) and transect exposition (NW, N, NE, E, SE, S, SW, W).

| **Year** | **Field** | **Transect type** | **Turning edge** | **Transect exposition** |
| --- | --- | --- | --- | --- |
| 2014 | 1 | Agricultural road  Field-to-field  Field-to-field  Hedgerow | No  Yes  Yes  No | NW  NE  NE  NW |
|  | 2 | Agricultural road  Hedgerow | Yes  No | SE  NE |
|  | 3 | Forest | No | SW |
|  | 4 | Forest | No | SE |
| 2015 | 1 | Forest  Forest | No  Yes | E  NE |
|  | 2 | Forest  Forest | No  No | SE  E |
|  | 3 | Forest  Forest | Yes  Yes | W  SW |
|  | 4 | Forest  Forest | Yes  No | NE  SE |
|  | 5 | Forest  Forest | Yes  Yes | E  NE |
|  | 6 | Forest  Forest | No  Yes | E  S |
| 2016 | 1 | Hedgerow | No | SE |
|  | 2 | Field-to-field  Kettle hole | No  No | SE  SE |
|  | 3 | Kettle hole | No | W |
|  | 4 | Hedgerow | No | NW |
|  | 5 | Hedgerow | No | NW |
|  | 6 | Field-to-field  Hedgerow | No  No | NW  SE |
|  | 7 | Field-to-field  Hedgerow | No  No | E  W |
|  | 8 | Kettle hole | No | E |
|  | 9 | Hedgerow | No | NW |
|  | 10 | Kettle hole | No | SE |
|  | 11 | Field-to-field  Kettle hole | No  No | E  E |
|  | 12 | Kettle hole | No | SE |
|  | 13 | Hedgerow | No | E |
| 2017 | 1 | Agricultural road  Hedgerow  Kettle hole | Yes  No  No | N  E  W |
|  | 2 | Field-to-field  Hedgerow | No  No | NW  NW |
|  | 3 | Field-to-field  Hedgerow  Kettle hole | No  No  Yes | NW  E  NE |
|  | 4 | Field-to-field  Kettle hole | No  No | SE  NW |
|  | 5 | Agricultural road  Kettle hole | No  No | W  E |
|  | 6 | Field-to-field  Kettle hole | No  No | S  NE |
|  | 7 | Agricultural road  Hedgerow | No  No | E  W |
|  | 8 | Field-to-field  Hedgerow  Kettle hole | Yes  Yes  No | E  W  N |
|  | 9 | Field-to-field  Hedgerow  Kettle hole | No  Yes  No | E  S  W |
|  | 10 | Agricultural road  Hedgerow  Kettle hole | No  No  No | SE  NW  SE |
|  | 11 | Field-to-field  Hedgerow  Kettle hole | Yes  No  Yes | E  NW  E |

Table S2: Type-II-analysis of variance table for linear mixed-effects model on crop yield as a function of year, turning edge and transect type *[yield ~ year + turning edge + transect type + turning edge : transect type, random= ~1| field / transect]*, bold font: significant (*P* < 0.05), normal font: not significant (*P* > 0.05). Results show that turning edges had only a marginal effect on our yield data.

|  | df | Chisq | p-value |
| --- | --- | --- | --- |
| Year | 3 | 2.3 | 0.5112 |
| Turning edge | 1 | 2.8 | 0.093 |
| Transect type | **4** | **29.4** | **< 0.001** |
| Turning edge:transect type | 4 | 5.8 | 0.218 |

Table S3: Post-hoc test with phia package (De Rosario-Martinez, Fox, & R Core Team, 2015) of the interaction term turning edge differences per transect type in the linear mixed-effects model of Table A2. Bold font: significant (*P* < 0.05), normal font: not significant (*P* > 0.05). Results show that the marginal overall effect of turning edges was dominated by the hedgerow data. At hedgerows, yields were higher at field borders with turning edges compared to those without turning edges (Fig. A1). Thus, we cannot conclude that yields measured at field borders with turning edges lowered yields compared to those without turning edges.

|  | Value | df | Chisq | p-value |
| --- | --- | --- | --- | --- |
| Field-to-field | -0.58 | 1 | 0.575 | 1.000 |
| Forest | 0.06 | 1 | 0.006 | 1.000 |
| Hedgerow | **-2.53** | **1** | **7.842** | **0.026** |
| Kettle hole | -0.41 | 1 | 0.208 | 1.000 |
| Agricultural road | 0.06 | 1 | 0.004 | 1.000 |

Table S4: Type-II-analysis of variance tables for linear mixed-effect model on crop yield as a function of year, orientation to the sun, transect type and distance [*yield ~ year + sun + transect type + Distance + transect type : sun, transect type : Distance, random= ~1| field / transect*], bold font: significant (P < 0.05), normal font: not significant (P > 0.05).

|  | df | Chisq | p-value |
| --- | --- | --- | --- |
| Year | 3 | 2.3 | 0.505 |
| Orientation to the sun | 1 | 0.1 | 0.760 |
| Transect type | 4 | 7.4 | 0.115 |
| Distance | **3** | **95.4** | **< 0.001** |
| Transect type:sun | 4 | 3.6 | 0.468 |
| Transect type:distance | **12** | **26.5** | **0.009** |
